# An artificially split class 3 intein

**DOI:** 10.17912/micropub.biology.000977

**Published:** 2023-09-22

**Authors:** Tia M. Ariagno, John S. Smetana, Christopher W. Lennon

**Affiliations:** 1 Department of Biological Sciences, Murray State University, Murray, Kentucky, United States

## Abstract

Inteins excise themselves from precursor polypeptides through protein splicing, joining N- and C-exteins with a peptide bond. Split inteins are expressed as separate polypeptides that undergo protein *trans* splicing (PTS). Here, we demonstrate PTS can be achieved using an artificially split class 3 intein. Because class 3 inteins use an internal initiating nucleophile near the C-extein junction, rather than the first residue of the intein, both catalytic nucleophiles are present on a single polypeptide. This results in a compact arrangement of catalytic nucleophiles for PTS compared to the standard arrangement for split class 1 inteins.

**Figure 1. Protein trans splicing of split M. smegmatis DnaBi1:**
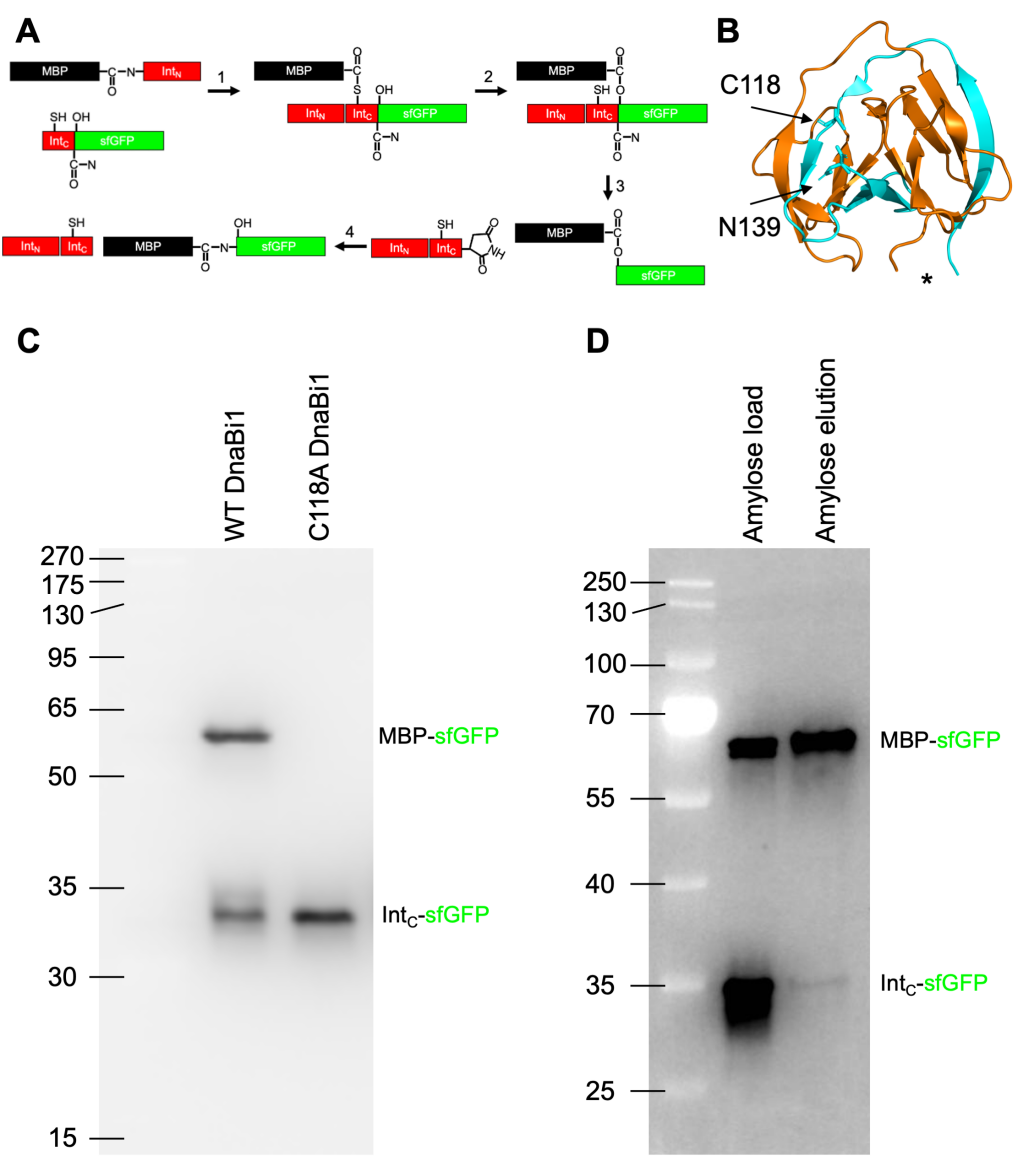
**A.** Mecahnisms of class 3 protein splicing with the Maltose Binding Protein (MBP) as the N-extein (black), superfolder GFP (sfGFP) as the C-extein (green), and the split intein (red). The peptide bonds between the N-extein and intein (prespliced) and N-extein and C-extein (spliced) are shown, as well as the side chains of the catalytic nucelophiles (Cys118 and Ser +1) and C-terminal intein asparagine. **B.** Structure of contiguous *Mycobacterium smegmatis* DnaBi1 generated using pymol from PDB 6BS8 (Kelley et al., 2018). Residues 1-88 are shown in orange and residues 101-139 are shown in cyan. Residues 89-100 are unstructured and labeled with an asterisk (site of artificial split is between residues 94-95). Inititating nucleophile (C118) and terminal asparagine (N139) are labeled and shown as sticks. **C.** MBP-DnaBi1 ^N^ and DnaBi1 ^C^ -sfGFP or DnaBi1 ^C^ C118A-sfGFP were co-expressed at 21ºC for 20 hours and in-gel fluorescence was used to measure GFP-containing products. **D.** MBP-sfGFP is both fluorescent and retained by amylose resin, demonstrating *trans* splicing between MBP-DnaBi1 ^N^ and DnaBi1 ^C^ -sfGFP. MBP-DnaBi1 ^N^ and DnaBi1 ^C^ -sfGFP were co-expressed at 16ºC for 20 hours prior to amylose resin pull-down.

## Description

**In** tervening pro **teins** (inteins) are translated within host proteins and excised via protein splicing (Paulus, 2000) . In this reaction, the intein rearranges the two flanking peptide bonds into one, in the process ligating the adjacent N- and C-exteins and escaping the host protein (ligated exteins) without a trace. The ability of inteins to perform this chemistry has been exploited for a variety of protein engineering applications.

The class 1 mechanism of protein splicing occurs in four steps (Mills et al., 2014) . First, an N-terminal intein residue (Cysteine or Serine) performs a nucleophilic attack on the peptide bond between the N-extein and intein, resulting in a (thio)ester linkage. Second, the first residue of the C-extein, a Cysteine, Serine, or Threonine, performs a second nucleophilic attack on the (thio)ester resulting from step 1, forming a branched intermediate. Third, the last residue of the intein, often a conserved asparagine, cyclizes to release the intein. Lastly, a peptide bond is formed between the N- and C-exteins through (thio)ester rearrangement. Class 2 and 3 splicing mechanisms have also been described. Class 3 inteins utilize a very similar strategy as class 1 inteins, except for the position of the initiating nucleophile that begins the splicing reaction - the initiating nucleophilic attack is carried out by an internal residue near the C-terminal intein-extein junction.

Inteins are particularly abundant in archaea and bacteria, but are also present in phages, viruses, and unicellular eukaryotes (Novikova et al., 2016) . Inteins can be full-length, meaning the intein has an internal homing endonuclease domain (HEN), which assists in the invasion of intein-free alleles and makes some inteins mobile genetic elements. Mini-inteins lack the HEN domain, but retain conserved splicing blocks necessary for splicing. Interestingly, some inteins have been split in nature and are synthesized as separate polypeptides that undergo protein *trans* splicing (PTS).

Inteins can be thought of as self-contained, single-turnover enzymes with a unique ability to break and remake peptide bonds at designated positions. As such, this ability has been exploited in numerous protein engineering applications including bioseparations, bioconjugation, biosensing, and protein cyclization (Wood and Camarero, 2014; Sarmiento and Camarero, 2019) . The impact of intein-based technologies on protein engineering has been, and continues to be, profound. Split inteins provide a unique avenue to control the protein splicing reaction by physical separation of intein fragments. Split inteins typically divide into larger N-intein (~100 residues) and smaller C-intein (~40 residues) domains (Eryilmaz et al., 2014; Aranko et al., 2014) . Upon reassociation, split inteins adopt a highly similar fold to *cis* splicing inteins.

Numerous split inteins using a class 1 splicing mechanism have been extensively engineered to improve rate, accuracy, and controllability of PTS (Eryilmaz et al., 2014; Aranko et al., 2014) , and only recently has there been the demonstration of *trans* splicing using alternative chemistries (Pinto et al., 2020; Hoffmann et al., 2021) . In this work, we artificially split the class 3 DnaB1 intein from *Mycobacterium smegmatis* (DnaBi1) and observe efficient splicing in *trans* . The major difference between this system and traditional split intein systems is that both the initiating and +1 nucleophiles, as well as conserved terminal asparagine of the intein, are on the same Int ^C^ -C-extein fragment. The expected mechanism of class 3 *trans* splicing is shown ( Figure 1A ).

The replicative helicase DnaB from *M. smegmatis* houses two inteins (DnaBi1 and DnaBi2). DnaBi1 is a mini-intein (139-residues) that utilizes a class 3 splicing mechanism, while DnaBi2 is a full-length intein that employs class 1 chemistry. The crystal structure of DnaBi1 shows a disordered loop between conserved splicing blocks, consistent with the expected Hedgehog/Intein (Hint) domain fold (Kelley et al., 2018) . This disordered loop, located ~40 residues from the intein-C-extein junction, is where most naturally *trans* splicing inteins are split. Several class 1 *cis* splicing inteins have been divided at this loop, resulting in engineered *trans* splicing (Aranko et al., 2014) . We sought to determine whether the class 3 *M. smegmatis* DnaBi1 could splice in *trans.* As the initiating nucleophile (C118) for *M. smegmatis* DnaBi1 is located after the disordered HEN loop, both catalytic nucleophiles would be housed on the C-intein-extein fragment. This design results in protein *trans* splicing with a compact arrangement of catalytic nucleophiles.

We introduced a split in DnaBi1 within the disordered HEN loop between residues 94 and 95 ( Figure 1B ) and measured *trans* splicing using a modified version of an established splicing reporter, MIG (Kelley et al., 2018) . In the MIG reporter ( **M** BP- **I** ntein- **G** FP), the intein (surrounded by 10 native extein residues) is flanked by the non-native exteins MBP (Maltose Binding Protein; N-extein) and superfolder GFP (sfGFP; C-extein). sfGFP-containing products can be detected using in-gel fluorescence following semi-native PAGE (Weinberger II and Lennon, 2021) . MBP-DnaBi1 ^N^ and DnaBi1 ^C^ -sfGFP were co-expressed from different promoters on the same plasmid in *E. coli* and sfGFP-containing products were measured. Two products were observed, the smaller of which is consistent in size with DnaBi1 ^C^ -sfGFP (32.8 kDa) and the larger with the expected product from *trans* splicing between MBP-DnaBi1 ^N^ and DnaBi1 ^C^ -sfGFP, MBP-sfGFP (69.7 kDa) ( Figure 1C ).

Previously, it was demonstrated that the initiating nucleophile C118 of DnaBi1 was required for splicing in *cis* (Kelley et al., 2018; Woods et al., 2020; Lennon et al., 2021) . We next tested whether this residue is also required for splicing in *trans* . As expected, and consistent with splicing between MBP-DnaBi1 ^N^ and DnaBi1 ^C^ -sfGFP, the larger product is completely absent when the C118A substitution is made on DnaBi1 ^C^ -sfGFP ( Figure 1C ). The requirement of both of C118 for splicing in *cis* and loss of the band consistent in size with MBP-sfGFP only when C118 is mutated strongly suggests protein *trans* splicing between MBP-DnaBi1 ^N^ and DnaBi1 ^C^ -sfGFP. It may be possible for the larger sfGFP-containing product to be a dimer of Int ^C^ -sfGFP (65.6 kDa), rather than MBP-sfGFP, as the initiating nucleophile C118 is free to form a disulfide. Additionally, while samples were not boiled to maintain sfGFP structure prior to semi-native SDS-PAGE, ß-mercaptoethanol was added to reduce any potential disulfide bonds and eliminate this possibility.

Finally, to further confirm that *trans* splicing had occurred, we reasoned that the larger sfGFP-containing band must also contain MBP. To determine whether the larger sfGFP-fluorescent product also contained MBP, we tested capture by amylose resin, which will pull-down any MBP-tagged products. Indeed, we found that the larger sfGFP-containing band was retained by the resin ( Figure 1D ). Taken together, these results clearly demonstrate the formation of MBP-sfGFP from coordinated MBP-DnaBi1 ^N^ and DnaBi1 ^C^ -sfGFP expression, a product that can only result from class 3 *trans* splicing.

Here, we show that a well-studied, small, conditionally-regulated (Kelley et al., 2018; Woods et al., 2020; Lennon et al., 2021) class 3 intein DnaBi1 from *M. smegmatis* can be artificially split for PTS applications. Due to the catalytic nucleophile arrangement of a class 3 intein, this design results in a compact arrangement of nucleophiles for *trans* splicing. As split inteins technologies based on class 1 inteins have been exceptionally useful in protein engineering, alternative intein chemistries may prove useful for certain applications.

## Methods

***Plasmids and strains***

Plasmids used in this study (pET-Duet1 MBP-DnaB1 Int _N_ / DnaB1Int _C_ -sfGFP; pET-Duet1 MBP-DnaB1 Int _N_ / DnaB1Int _C_ -sfGFP C118A) were synthesized (Genscript) and were transformed into *Escherichia coli* BL21(DE3) for protein expression.

***Protein expression***

Cells were grown in LB with 100 µg/mL ampicillin at 37ºC to mid-log (OD _600_ ~0.5), and protein expression was induced by addition 1 mM Isopropyl β- d-1-thiogalactopyranoside (Gold Bio). Protein expression proceeded for either 20 hours at either 16ºC or 21ºC after which cells were pelleted by centrifugation.

***Amylose resin purification***

Following protein expression (MBP-DnaBi1 ^N^ or MBP-DnaBi1 _STOP_ ), cells were resususpened in 20 mM Tris (pH 7.5), 200 mM NaCl (Amylose Column Buffer) with 1X HALT Protease Inhibitor Cocktail (Thermo Scientific), and lysed by sonication. Crude lysate was centrifuged at 20,000 x g, 4ºC and supernatant was applied to Amylose Resin (New England Biolabs) equilibrated in Amylose Column Buffer, washed with ~20 column volumes of Amylose Column Buffer, and eluted with Amylose Column Buffer plus 10 mM maltose.

***SDS-PAGE***

For in-gel fluorescence, cells were resuspended in 50 mM Tris-HCl (pH 8.0), 20 mM NaCl, 10% glycerol and lysed by sonication. Insoluble material was removed by centrifugation and soluble lysate was mixed with 4X Laemmli Sample Buffer with 4% ß-mercaptoethanol (Bio-Rad). Samples were separated using 8-16% Tris-Glycine TGX gels (Bio-Rad). Samples were not heated to maintain sfGFP structure and fluorescent products were detected with an Amersham Imager 680 (GE Healthcare).

## Reagents

**Table:** 

**Table:** 

**Strain**	**Genotype**	**Source**
*Escherichia coli* BL21(DE3)	*fhuA2 [lon] ompT gal (λ DE3) [dcm] ∆hsdSλ DE3 = λ sBamHIo ∆EcoRI-B int::(lacI::PlacUV5::T7 gene1) i21 ∆nin5*	NEB
**Plasmid**	**Description**	**Source**
pET-Duet1 MBP-DnaB1 Int _N_ /DnaB1Int _C_ -GFP	Plasmid encoding for DnaB1 intein with MBP N-extein and GFP C-extein.	GenScript
pET-Duet1 MBP-DnaB1 Int _N_ /DnaB1 Int _C_ -GFP C118A	Plasmid encoding for DnaB1 intein with MBP N-extein and GFP C-extein. Has a substitution on C118A.	GenScript
